# Supplementary material for: Inhibition of MCP1 (CCL2) Enhances Antitumor Activity of NK Cells Against HCC Cells Under Hypoxia
Source: Int J Mol Sci. 2025 May 20;26(10):4900. doi: 10.3390/ijms26104900 (PMC12111856; doi:10.3390/ijms26104900)
Supplement: Supplementary file 1 [file ijms-26-04900-s001.zip › ijms-3603533-supplementary.pdf]

Supplementary Figure S1

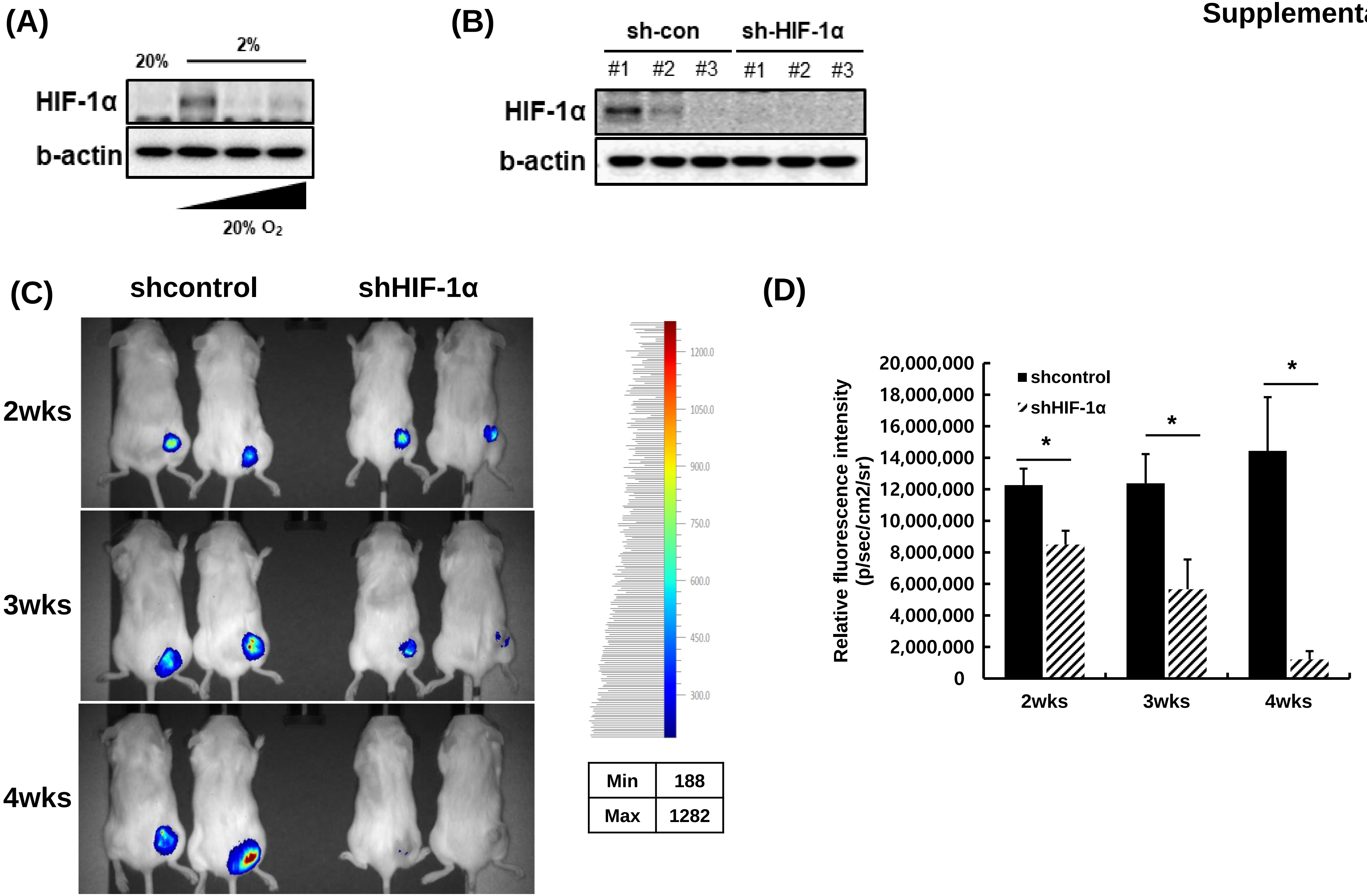

Supplementary Figure S2

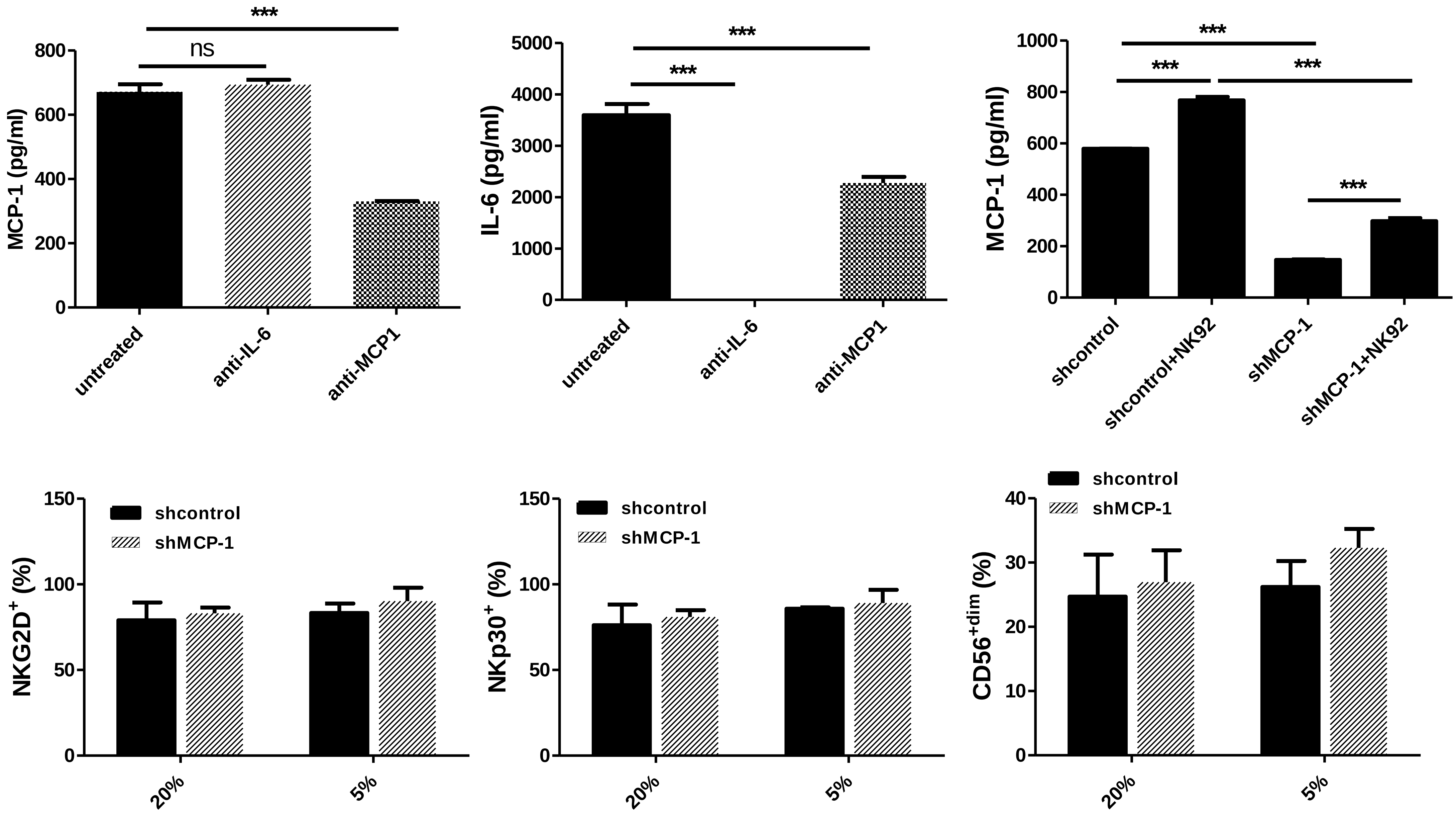

Supplementary Figure S3

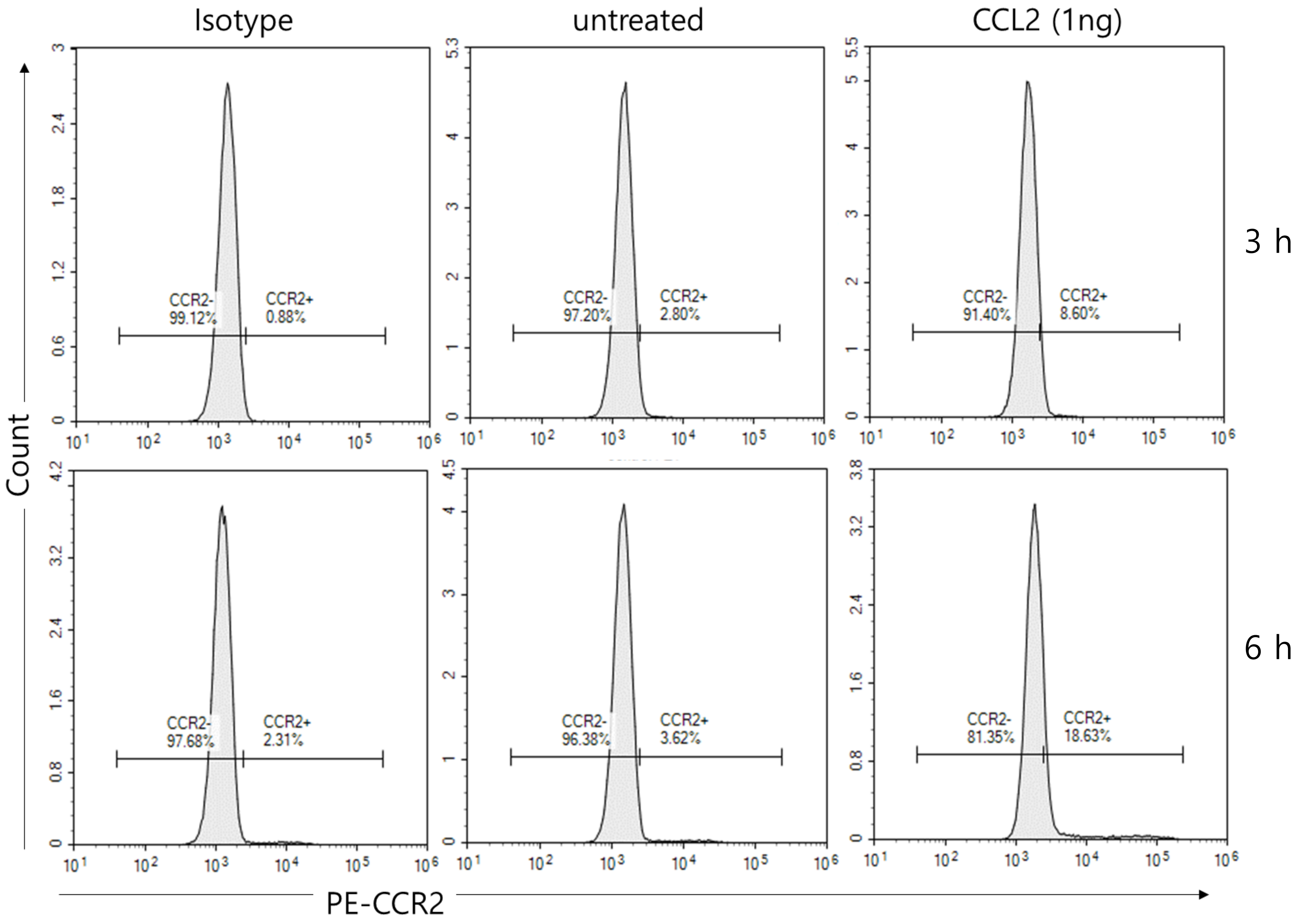

Figure S1. (A–D) Effect of HIF-1α expression on HCC tumor growth using xenograft mouse.

Figure S2. Decreased levels of MCP1 by treatment with anti-IL-6 antibody in HCC cell culture. *** $p < 0.0001$, ns–not significant.

Figure S3. CCR2 expression on the surface of NK-92 by treatment with CCL2.
